# Reconstruction of the trachea and carina: Surgical reconstruction, autologous tissue transplantation, allograft transplantation, and bioengineering

**DOI:** 10.1111/1759-7714.14315

**Published:** 2022-01-13

**Authors:** Jianghao Ren, Yuanyuan Xu, Guo Zhiyi, Ting Ren, Jiangbin Ren, Kan Wang, Yiqing Luo, Mingyang Zhu, Qiang Tan

**Affiliations:** ^1^ Department of Thoracic Surgery Shanghai Chest Hospital, Shanghai Jiaotong University Shanghai China; ^2^ Huai'an First People's Hospital, Nanjing Medical University Huai'an Jiangsu China; ^3^ The 4th Affiliated Hospital of Harbin Medical University Harbin China

**Keywords:** bioengineering, regenerative medicine, surgery, tracheal reconstruction, transplantation

## Abstract

There have been significant advancements in medical techniques in the present epoch, with the emergence of some novel operative substitutes. However, the treatment of tracheal defects still faces tremendous challenges and there is, as yet, no consensus on tracheal and carinal reconstruction. In addition, surgical outcomes vary in different individuals, which results in an ambiguous future for tracheal surgery. Although transplantation was once an effective and promising method, it is limited by a shortage of donors and immune rejection. The development of bioengineering has provided an alternative for the treatment of tracheal defects, but this discipline is full of ethical controversy and hindered by limited cognition in this area. Meanwhile, progression of this technique is blocked by a deficiency in ideal materials. The trachea together with the carina is still the last unpaired organ in thoracic surgery and propososal of a favorable scheme to remove this dilemma is urgently required. In this review, four main tracheal reconstruction methods, especially surgical techniques, are evaluated, and a thorough interpretation conducted.

## Graphical abstract

Tracheal and carinal reconstruction are still challenges in regenerative medicine although medical techniques have achieved significant advancements in the present epoch.

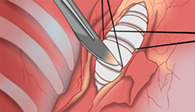

## INTRODUCTION

As a channel connecting the larynx and bronchi, the trachea extends from the sixth to the seventh cervical vertebrae (larynx) to the fourth–fifth thoracic vertebrae reaching the carina, which is then divided into the left and right main bronchi.

The normal trachea in adults is ~10 to 14 cm in length. It is not only a pathway for ventilation, but also has the function of removing foreign bodies, natural immunity, and so on. However, there are still significant challenges in the reconstruction of the trachea and carina due to their special structural features (transverse flexibility, longitudinal rigidity, ciliated epithelial cell defense mechanisms and so forth) and complicated adjacent anatomies. The most common causes for tracheal diseases include benign airway stenosis, lung or tracheal tumors, local patchy defects, and tracheal rupture (Table 1).

**TABLE 1:** Pathogenesis of tracheal diseases requiring surgery

Benign airway stenosis	Benign airway stenosis is the most common cause, including transmural defect of the ventral trachea after tracheotomy, wall softening, stenosis after intubation, idiopathic inflammation, and stenosis^13^
Lung tumor	Tumor in the lung would invade the trachea. Under this circumstance, resection of partial lung tissues on the side of lesion should always be performed
Primary tracheal tumor	Primary tracheal tumors have an extremely low incidence in adults and almost 90% of them are malignant; the proportion in children is about 30%. Those individuals suffering from primary tracheal tumors, with morbidity of about 1 in one million, account for 0.2% in those with respiratory tumors, and 0.02%–0.04% in all registered cases with malignancies. The most common pathological type is squamous cell carcinoma (SCC, 36%–45%) and adenoid cystic carcinoma (ACC, 31%–40%)^11^, ^12^, ^30^, ^65^, ^66^
Local patchy defect or rupture of trachea	Trachea/bronchus‐pleural fistula, trachea/bronchus‐esophagus fistula, trauma, and respiratory injuries

Up to now, end‐to‐end anastomosis followed by tracheal resection is still the gold standard surgical repair technique, but there are some limitations in this method. For example, anastomotic tension will increase when the length of resection exceeds 4–6 cm, which would cause severe anastomotic complications. There are some alternatives when end‐to‐end anastomosis is inaccessible: transplantation reconstruction (including allograft transplantation and xenograft transplantation), prosthetic implantation, and tissue‐engineering procedures. Nevertheless, all those operations are in dispute, and further investigations and practice are required to reach a consensus. In this review, the focus is placed on the interpretation and summary of the four main surgical reconstruction methods for tracheas.

## SURGICAL RECONSTRUCTION

### Development history

Tracheotomy has been widely applied prior to the emergency management of the tracheal reconstruction, but surgery was soon suspended due to the high morbidity and mortality, as well as the limited surgical techniques. In 1946,^1^ Belsey utilized and evaluated the stainless steel wire suture technique in thoracic surgery and systematically introduced tracheal surgery into the thoracic operation four years later in 1950.^2^ Since then, tracheal surgery began to development rapidly. Thomas performed the first tracheal resection and anastomosis in 1956, which promoted tracheal reconstruction to a new height.^3^ In 1950, Abott reported a case of early carinal resection,^4^ which marked that surgery had begun to develop in a complicated direction and aroused the subsequent reflection on carinal reconstruction. In 1957, Barclay et al. carried out the first carinal resection and reconstruction by forming a neo‐carina.^5^ Subsequently, many articles and reports appeared, such as the first carinal pneumonectomy in 1958 performed by Matthes, in which the tracheal end was anastomosed to the left main bronchus.^6^ Grillo shared his experiences in carinal resection and primary reconstruction on 36 patients from 1962 to 1981, in which there was miraculously only a 13% mortality rate.^7^ Those investigators promoted the advancement of tracheal surgery, thus leading to a marvelous age during the 1960s when this discipline achieved rapid development.^8^ In 1990, the removal rate of tracheal tumors had reached 63%, squamous cell carcinoma (SCC) 63%, adenoid cystic carcinoma 75% and other tumors 90%.^9^ Nowadays, these techniques in tracheal resection and carinal reconstruction have gradually matured, based on which some emerging technologies have also been developed, including transplantation and reconstruction, tissue engineering, and so forth.

### Surgical techniques

#### Short segment tracheal resection

**FIGURE 1:**
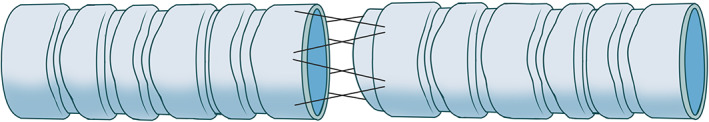
The gold standard for clinical tracheal resection and reconstruction of end‐to‐end anastomosis

From the 1960s to 1970s, an empirical conclusion was drawn with an increase in the number of tracheal operations and development of technologies; for example, end‐to‐end anastomosis (Figure 1) between both residual ends became the primary method and the golden standard for clinical tracheal resection and reconstruction.^10^, ^11^ The normal length of an adult trachea is ~10–14 cm. In theory, the resectable length is 50% for adults and one‐third for children, which is demonstrated in many previously reported studies.^12^, ^13^ However, the specific resection length varies individually according to the specific conditions of patients. In most cases, apparent anastomotic tension will not appear when the excised trachea does not exceed 4–6 cm, which would in turn cause low mortality and morbidity.^14^, ^15^, ^16^ It has been confirmed in the literature that the morbidity is 10 and 32%, respectively for the resection length of tracheas <4 cm and >4 cm.^17^

#### Long segment tracheal resection (narrow length > 2/3)

**FIGURE 2:**
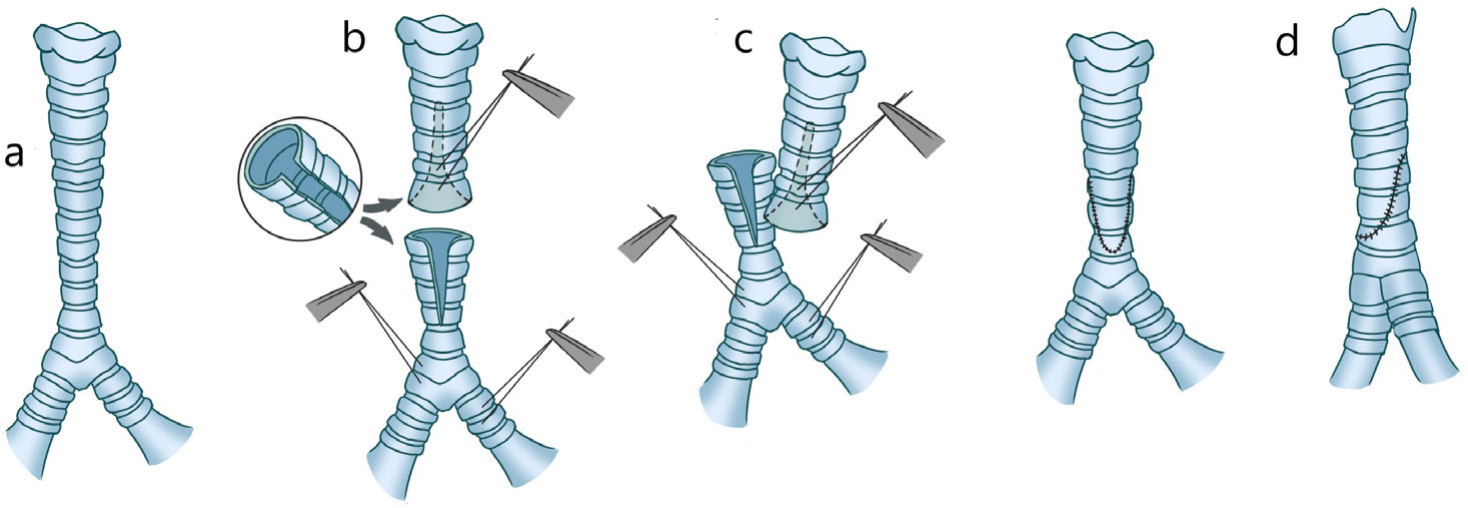
The slide tracheoplasty. (a) Long‐segment stenosis is identified in the middle part of the trachea transversely. (b, c) The resection should be performed transversely at the midpoint of the tracheal stenosis, the proximal and distal trachea are bifurcated and the two bifurcated pieces are subsequently overlapped. (d) Slide these two parts towards each other and finally perform the suture (this image is reprinted and extracted from Grillo)^22^

Slide tracheoplasty has gradually replaced patch technology, allotransplantation, and prosthesis replacement, and it is widely accepted to be the standard therapeutic measure in congenital airway stenosis or pediatric airway stenosis with the development of surgery. Slide tracheoplasty (Figure 2) was first reported in the treatment of high long‐segment tracheal stenosis in children in 1989, when it was performed to release the narrow airway without resecting the trachea, which caused a dramatic sensation.^18^ In subsequent reports, the effectiveness of this operation was confirmed.^19^, ^20^ Excessive resection will block the postoperative remodeling of tracheas, although, in theory, the upper limit is one third in children. In a slide tracheoplasty, the midpoint of the tracheal stenosis is cut transversely, then the proximal and distal tracheas are bifurcated; subsequently, the two bifurcated pieces are overlapped, and finally remodeled.^18^ In 2018, Redmann et al. reported the successful application of sliding tracheoplasty in the treatment of long tracheal stenosis in adults between 2011 and 2017 for the first time, with a reported complication rate of 32%.^21^ Figure 8 deformity is a common complication in the pediatric slide tracheoplasty while it is rare in adults due to their wider and stiffer airway.^21^ However, it is accustomed to adopting allotransplantation or tissue engineering rather than slide tracheoplasty in clinical practice for long‐segment stenosis.

#### Proximal laryngotracheal resection

The complicated anatomy of the laryngotrachea, important adjacent organs and the existence of thyroid cartilage as well as cricoid cartilage have always made high laryngotracheal reconstruction the main challenge in thoracic surgery. Normally, end‐to‐end anastomosis is still the first choice only if the resection length is not likely to induce obvious complications. Nevertheless, laryngotracheal stenosis is more common in children and infants, and it is often caused by congenital diseases. Therefore, the selection of end‐to‐end anastomosis will certainly require the partial resection of tracheal tissue, which may affect the postoperative reconstruction of tracheas. For that reason, it is necessary to conduct a comprehensive analysis of the resection length. Clinically, the preferred choice is laryngotracheoplasty, in which autologous or allograft tissue is used for repair. Certainly, slide tracheoplasty would be given priority if the length of the lesions is too long.

#### Distal carinal reconstruction

##### Complete carinal resection

###### Carinal excision of limited length (within a minor range)

**FIGURE 3:**
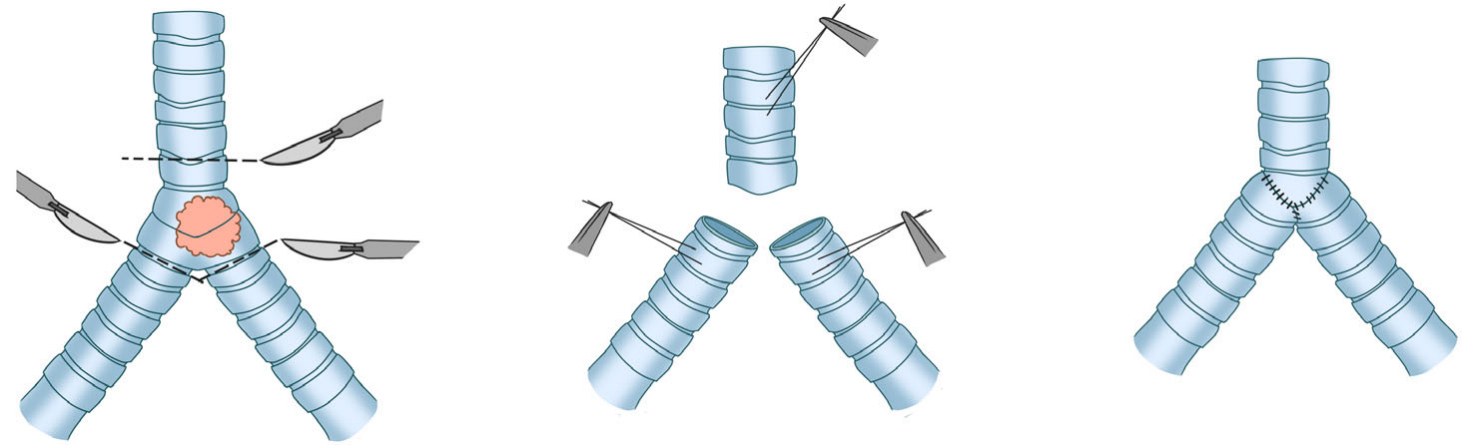
Anastomose the two main bronchi and trachea to form a new carina

Anastomosis of the left and right main bronchi is recommended to create a new carina, and the new carina should be anastomosed to the tracheal stump. However, due to the fact that the proximal movement of the newly formed carina is limited by the aortic arch, most of the airway movement in the reconstruction process depends on the trachea.^23^ On the one hand, when the resection range is large, it would be difficult to move the new carina formed by the suture of the medial wall of the left and right main bronchi upward due to the limitation of the aortic arch. On the other hand, the cross anastomosis of these two main bronchi and trachea is often poorly matched, which may affect the blood supply. Therefore, this technique is rarely adopted in clinical practice in these circumstances Figure 3.

###### Resection range‐extended but within the limitation (trachea to main bronchi is 4 cm)

Considering the tension of anastomosis, end‐to‐end anastomosis of the left main bronchus to the trachea is more commonly performed, followed by an end‐to‐side anastomosis of the right main bronchus into the side of the trachea. The inferior hilar release is supplementary to reduce the anastomosis tension and to mobilize the airway. When the trachea is to be anastomosed to the left main bronchus, the scope of airway resection should be limited to <4 cm, which is conducive to minimizing the risk of anastomotic complications.^4^, ^24^ When the distance between the trachea and the left main bronchus (the length of the resected airway) is more than 4 cm, this reconstruction may lead to excessive anastomotic tension, even after performing the release operation. This is mainly caused by the relative stiffness of the left bronchus and the limited movement of its head by the aortic arch. However, it is not suitable to apply these restrictions in the end‐to‐end anastomosis between the trachea and the right main bronchus which would move widely and get completely released through the complete releasing of the right hilum.^23^

**FIGURE 4:**
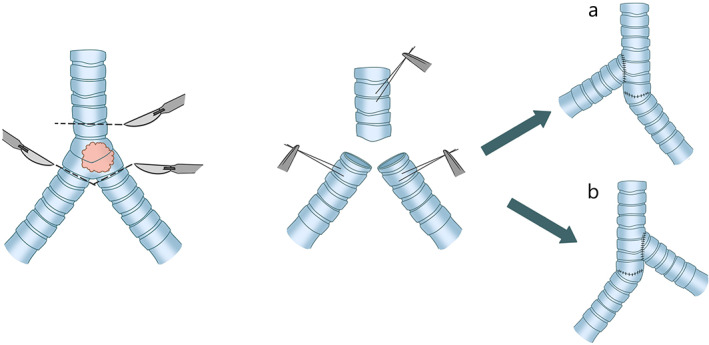
(a) End‐to‐end anastomosis of the left main bronchus to the trachea, followed by end‐to‐side anastomosis of the right main bronchus into the side of the trachea. (b) End‐to‐end anastomosis of the right main bronchus to the trachea, combined with reimplantation of the left main bronchus into the side of the trachea

It is rare for the contrary method of reconstruction (end‐to‐end anastomosis of the right main bronchus to the trachea) combined with reimplantation of the left main bronchus into the side of the trachea to be adopted, partly because of difficult surgical exposure Figure 4.

###### Scope of resection continues to expand (with the distance of more than 4 cm)

**FIGURE 5:**
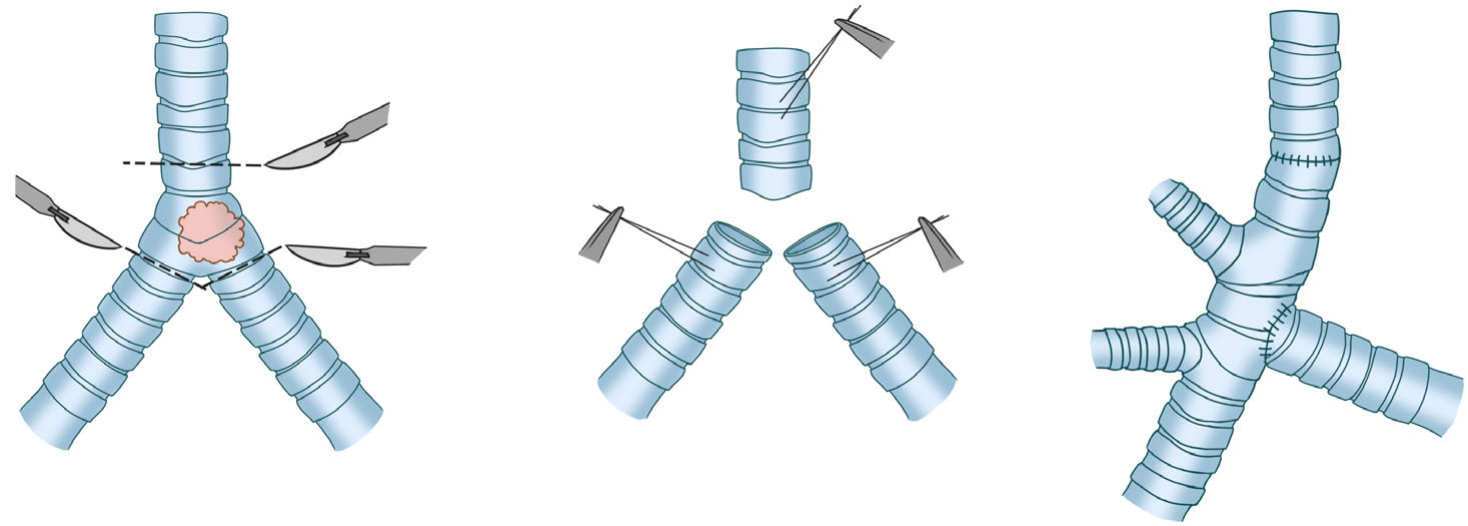
The end‐to‐end anastomosis of the right main bronchus to the trachea and the end‐to‐side anastomosis of the left main bronchus into the intermediate bronchus

It is preferable to take the end‐to‐end anastomosis of the right main bronchus to the trachea with the assistance of hilar release and the end‐to‐side anastomosis of the left main bronchus into the intermediate bronchus for decreasing the potential anastomotic tension. This operation can be performed safely only if these main bronchi (especially the right bronchus) remains long enough after carinal resection Figure 5.^25^

##### Incomplete carinal resection

**FIGURE 6:**
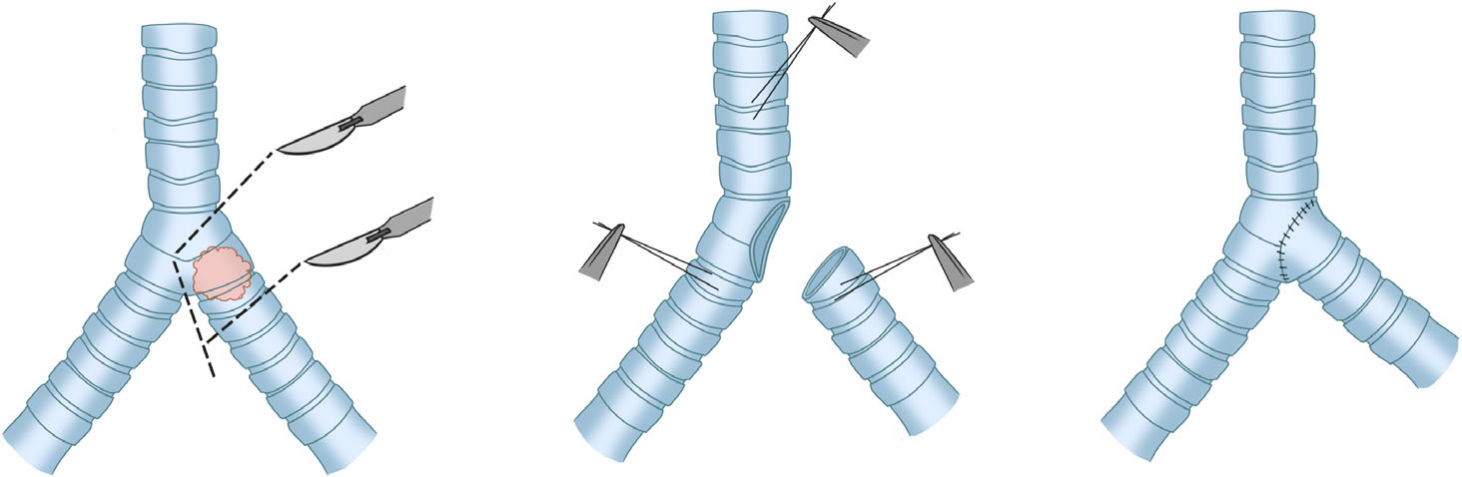
Suture the main bronchus directly to the incomplete carina

Partial carinal resection can be performed in cases where the lesion mainly involves the right or left main bronchus but the carina is less involved. The affected main bronchus or trachea can be resected separately to maintain the continuity between the trachea and contralateral bronchus, and then the separated bronchus can be sutured directly to the defect of the tracheobronchial wall caused by the resection Figure 6.

##### Lobectomy

In the process of carinal reconstruction, the involved upper lobe of the right lung or the middle lobe of the right lung would be resected at the same time. When the right upper lobe is resected, end‐to‐side anastomosis of the intermediate bronchus into the side of the trachea is usually performed by means of hilar release. However, there is a hidden risk that the intermediate bronchus would be pushed to the side of the trachea for anastomosis, which may induce airway necrosis and stenosis. These complications are caused by the angle, cutoff, and tension of the anastomosis.

Therefore, it is safer to reimplant the right intermediate bronchus into the left main bronchus, which contributes to reducing the anastomotic height and approaching the physiological state. Similarly, when it comes to the right middle lobe, the optimal anastomosis position of the residual right lower lobe bronchus is on the lateral wall of the left main bronchus.^23^ Chen proposed a novel technique of tracheal reconstruction with an autologous bronchial pedicle flap. This new operation was utilized to excise lung cancer originating from the right main bronchus and extending along the lateral wall of the lower segment of the trachea without involving the carina. The uninvaded left wall of the right main bronchus was sutured to the defect of the right wall of the trachea as an autologous pedicle flap together with the residual right middle and lower lobes. Unfortunately, no further results and prognosis have been reported.^26^

**FIGURE 7:**
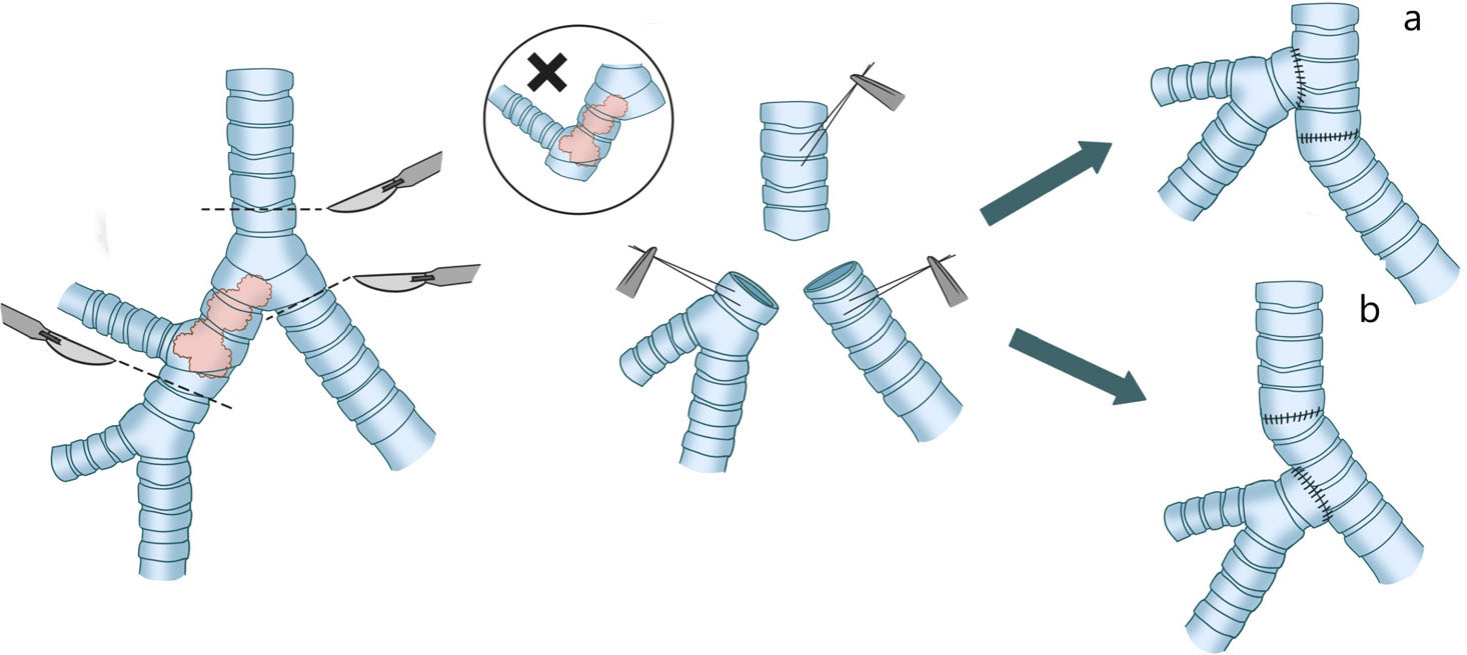
Carina reconstruction involving the right upper lobe resection. (a) End‐to‐side anastomosis of the intermediate bronchus into the side of the trachea. (b) Reimplantation of the right intermediate bronchus into the left main bronchus

In terms of the left lobe, a left thoracotomy has always been a challenge in carinal surgery, due to the fact that the existence of the aortic arch would not only deepen the surgical field but also restrict the exposure of the carina, trachea, and right main bronchus. It has been reported in the literature that left lobectomy is mainly performed through the left chest wall, but fewer carinal reconstructions are performed due to the difficulty of the procedure Figure 7.^17^

##### Pneumonectomy

**FIGURE 8:**
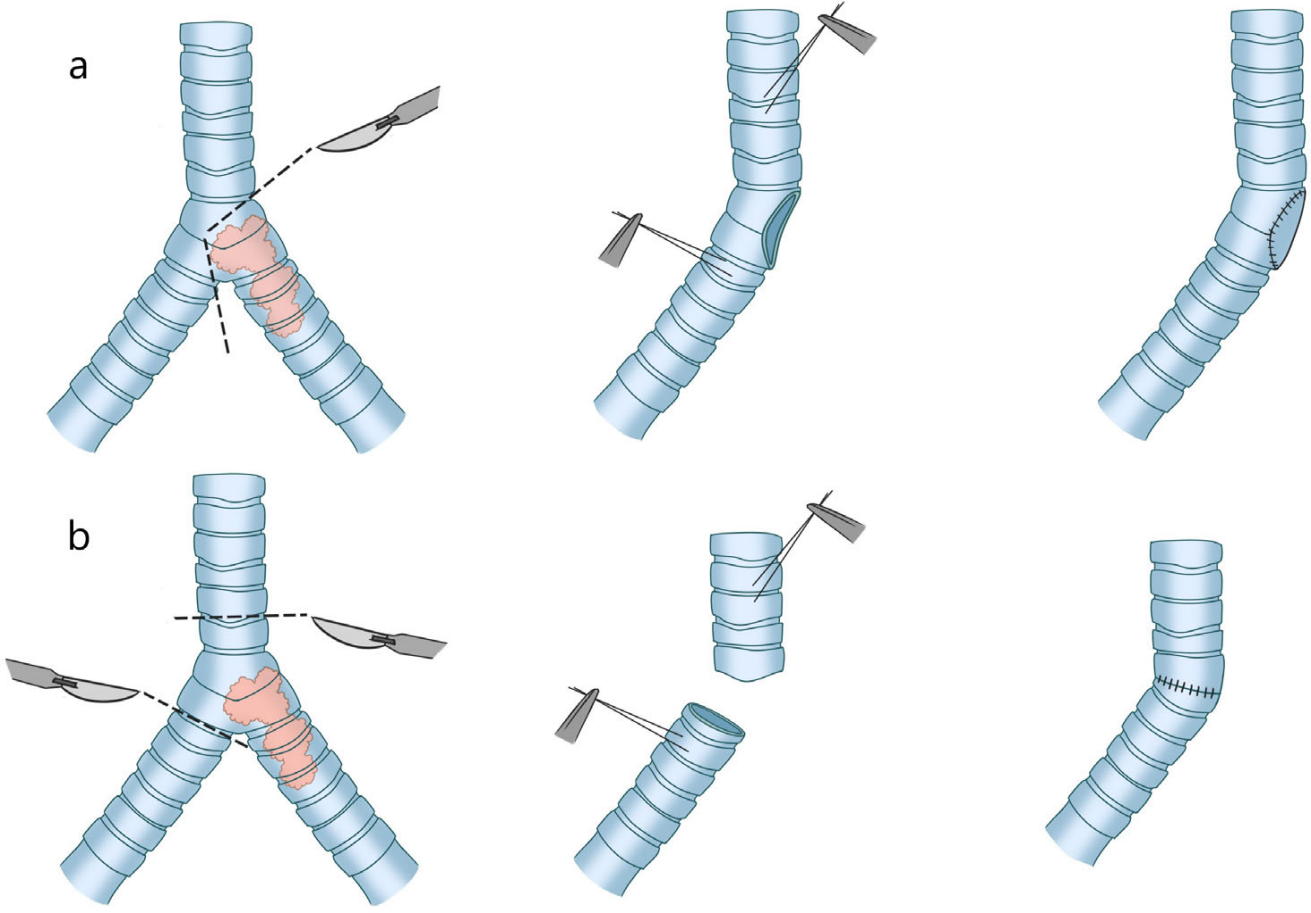
Carina reconstruction involving pneumonectomy

When there is extensive endobronchial involvement, both lungs are destroyed by obstruction, hilar vascular involvement, and the invasion of tumors into the whole lung. Therefore, the remaining lung cannot be preserved, and hence carinal pneumonectomy may be required. The carinal pneumonectomy is performed primarily to achieve tension‐free anastomosis and ensure that the patients can survival with remaining lungs. Therefore, a thorough evaluation of pulmonary function through various indicators before the operation is required in order to reduce postoperative morbidity and mortality. End‐to‐end anastomosis of the remaining contralateral main bronchus and trachea is performed after the pneumonectomy. It is recommended that the distance between the lower trachea and contralateral main bronchus should not exceed 4 cm.^24^, ^27^ If the infiltration depth of lesions into the trachea does not exceed 2 cm to the distal trachea and 1.5 cm to the proximal main bronchus, an extensive resection and tension‐free anastomosis can be achieved Figure 8.^28^, ^29^

### Attention in this reconstruction

#### Positive resection margin

It is predicted that nearly 40% of patients will have a positive resection margin and the majority would suffer from tumors derived from salivary glands, such as adenoid cystic carcinoma that has a tendency to diffuse along the perineurium to be away from the actual general margin of the tumor.^30^ As for those patients with a positive margin, adjuvant radiotherapy should be considered once the incision heals, as suggested by Gaissert et al. However, as per the opinions of Mitchell et al., the positive resection margin would not affect the surgical outcome and prognosis and the recurrence in patients with a negative margin of up to 22%.^31^

#### Anastomotic measures

Limited dissociation should be performed during anastomosis in an attempt to maintain an abundant blood supply at the anastomotic site. It is agreed that the dissociated extension should not exceed 2 cm and the distance between the right and left main bronchi at the anastomotic site should be more than 1–2 cm to avoid necrosis of the medial bronchial wall.^17^, ^23^, ^27^ The anastomotic site should be wrapped locally with pedicle flap or some blood‐supply tissue, such as a pleural flap, pericardial flap, intercostal muscles, and omentum flap. Meanwhile, keeping a certain distance from adjacent vessels to prevent postoperative bronchovascular fistula is required. However, before that, air‐ and fluid‐tightness should be confirmed. Finally, for the end‐to‐side anastomosis, the airway opening should be completely within the cartilage and away from the membrane in order to provide rigidity.

#### Principle and objective

Free‐tension anastomosis, abundant blood supply, well‐matched anastomosis, reconstruction of fluid dynamics and rigidity according to the physiological situation. Decrease postoperative morbidity and mortality as much as possible.

#### Adjuvant chemotherapy

There are some controversies over adjuvant chemotherapy in tracheal surgery. Shin et al. have argued that adjuvant chemotherapy is recommended in the following situations: microscopic residual tumor, involving mediastinal structures, and mediastinal lymph node metastasis.^32^ However, those patients who receive adjuvant therapies especially chemotherapy are at a higher risk of complications due to relevant tissue ischemia.^33^

#### Impact of suture techniques

In 2015, Palade et al. conducted a comparison between two suture techniques: a running suture at the membranous part and an interrupted suture for the rest of the circumference versus a telescoping continuous suture. The results showed that there was no significant difference in postoperative pulmonary complications, anastomotic complications, and mortality.^34^

### Postoperative complications

There are many factors affecting postoperative complications. The most important factor is the length of resection, followed by the tension of the anastomosis. Excessive tension of the anastomosis will lead to dehiscence and even fistula. The destruction of the blood supply, the selection of the surgical techniques, and preoperative induction therapies can also increase the incidence of postoperative complications. The incidence of complications would increase from 6.7% to 13%–24% after preoperative induction therapies.^27^ In this operation, it is necessary to achieve accurate anastomosis and remain in the areas with abundant blood supply to avoid cutoff of vessels. The summary of results from some relative studies are presented in Table 2. As per the reporting results, the mortality of tracheal reconstruction ranges from 1.2% to 12.7%.

**TABLE 2:** Outcomes in tracheal and carinal reconstruction

Author	Online year	Number	Constitution	Mortality	Morbidity	Anastomotic complications^a^	ARDS	Respiratory^b^	Cardiopulmonary events^c^	Empyema/hemothorax	Pneumonia	Lung oedema	Atelectasis
Mitchell et al.^23^	1998	134	Carinal resection^52^	17 (12.7%)	52 (38.8%)	23 (17.2%)	10 (7.5%)	0	21 (15.7%)	3 (2.2%)	11 (8.2%)	0	0
			Right carinal pneumonectomy^44^										
			Left carinal pneumonectomy^13^										
			Carinal plus lobar resection^14^										
			Others^11^										
Roviaro et al.^67^	2000	49	Right sleeve pneumonectomy^48^	4 (8.2%)	8 (16.3%)	1 (2%)	–	2 (4.1%)	2 (4.1%)	1 (2%)	1 (2%)	0 0	
			Left tracheal sleeve pneumonectomy^1^										
Wright et al.^35^	2004	901	Post intubation tracheal stenosis (589)	11 (1.2%)	164 (18.2%)	81 (9%)	–						
			Tumor (208)										
			Idiopathic laryngotracheal stenosis(83)										
			Tracheoesophageal fistula^21^										
Zhou et al.^17^	2006	84	Carinal resection and reconstruction	2 (2.4%)	32 (38.1%)	–			16 (19.1%)	1 (1.2%)	9 (10.2%)	0	6 (7.1%)
Jiang et al.^68^	2009	41	Carinal resection and reconstruction	1 (2.4%)	33 (80.5%)	3 (7.3%)	0	5 (12.2%)	12 (29.3%)	0	5 (12.2%)	0	8 (19.5%)
Milman et al.^69^	2009	64	Sleeve resection	2 (3.1%)	29 (45.3%)	2	0	0	8 (12.5%)	0	20 (31.25%)		
Gómez‐Caro et al.^70^	2011	58	Bronchal reconstruction	2 (3.4%)	20 (34.5%)	4 (6.9%)	1 (1.7%)	17 (29.3%)	3 (5.2%)	3 (5.2%)	6 (10.3%)	0	8 (13.8%)
Shin et al.^32^	2014	30	Carinal sleeve right pneumonectomy^17^	12 (40%)	11 (36.7%)	1 (3.3%)	3 (10%)	0	4 (13.3%)	3 (10%)	0	0	0
			Carinal sleeve left pneumonectomy^2^										
			Carinal sleeve left pneumonectomy^9^										
			Airway only^2^										
Palade et al.^34^	2015	60	Sleeve resection for lung cancer^60^	5 (8.3)	39 (65%)	6 (10%)	3 (5%)	11 (18.3%)	0	1 (1.7%)	13 (21.7%)	2 (3.3%)	3 (5%)
Costantino et al.^4^	2018	45	Carinal resection and reconstruction	3 (6.7%)	26 (57.8%)	6 (13.33%)	3 (6.7%)	5 (11.11%)	10 (22.22%)	0	11 (24.4%)	0	0

^a^ Anatomic complications: fistula/stenosis/local recurrence/dehiscence/ischemia.

^b^ Respiratory complications:Early respiratory failure/respiratory insufficiency.

^c^ Cardiopulmonary events: Embolism/arrhythmia.

Anastomotic complications mainly include granulation tissue, dehiscence due to excessive tension, and restenosis. In most cases, it is inevitable to generate granulation tissue, for the reason that there is a potential anastomotic fault that may allow the granulation to grow into the lumen. Generally, mild granulation tissue can be removed by fiberoptic bronchoscopy in the postoperative stable stage. Excessive granulation is always combined with such clinical symptoms as wheezing. If necessary, the steroid hormone is required. Resurgery or stent implantation would be adopted in rare cases. As the most severe and fatal complication, anastomotic rupture can be caused by excessive tension and occurs when the anastomotic tension surpasses the theoretic value (1000 g). However, actually, the incidence of this complication depends on individuals.^35^ Under the condition of emergencies, a bedside tracheotomy is required to fix the airway or a distal endotracheal tube to prepare for a second operation.

Some surgeons have summarized several measures to reduce the tension. The most common method is to bend the patient's neck, through which the trachea is mobilized to the mediastinal structure. However, this operation can only reduce the anastomotic tension to a limited extent due to the limited flexion of the neck. Incising and exposing the anterior tracheal plane would increase the tracheal mobility without affecting the lateral blood supply. Hence, it is generally applied in carinal reconstruction. The most effective technique is the inferior hilar release, in which it is required to incise inferior pulmonary ligament, make a u‐shaped incision at the pericardium below the hilum with an intrapericardial division of the raphe, and perform the extension between the inferior pulmonary vein and inferior vena cava. The complete hilar release should be taken into account if necessary. The presence of scar tissue at the anastomotic site may lead to restenosis, which can also be caused by acute recurrence after incomplete resection and by ischemia, inflammation, etc.

Anastomotic complications always occur in the late stage. Acute respiratory distress syndrome (ARDS) is the most serious complication in the early postoperative period, and may be caused by systemic inflammatory response syndrome (SIRS), improper use of ventilators or excessive infusion after the operation. In order to avoid ARDS, appropriate respiratory techniques should be adopted during the operation to avoid a high concentration of oxygen, reduce repeated pulmonary collapse and redilation and avoid hypoxic contraction of pulmonary vessels during low ipsilateral perfusion or high contralateral perfusion. It is significant to maintain sufficient oxidation, conduct adequate pain control, and avoid excessive expansion of the remaining lung after the operation. When ARDS occurs, tracheotomy should be performed immediately, which contributes to the secretion removal.^25^ Early respiratory failure usually occurs 1–2 days after the operation and those patients usually present with diffuse pulmonary interstitial infiltration, which would cause high mortality. Even if a series of conservative measures such as diuresis, antibiotics, hormones, and auxiliary ventilation have been taken, the situation may continue to deteriorate quickly.

Fistula is also a common complication. Tracheobroncho‐esophageal fistula has a relatively better prognosis compared with tracheal/bronchopleural fistula (BPF), and it is easy to diagnose and treat. Roviaro et al. found that preoperative high‐dose radiotherapy would increase the risk of BPF.^36^ The postoperative mechanical ventilation is a risk factor for BPF.^37^ When a fistula occurs, some positive therapeutic strategies should be carried out promptly, including draining instantly, infusing antibiotics, and repairing the defect as much as possible.

Intraoperative injuries of the recurrent laryngeal nerve always present with hoarseness, dysphagia, swallowing dysfunction, and laryngeal edema. These injuries can be relieved by bedside bronchoscopy. There are some complications not mentioned above, such as arrhythmia especially atrial fibrillation, pneumonia, and empyema.

## AUTOLOGOUS TISSUE TRANSPLANTATION

When surgical reconstruction is not feasible, especially when end‐to‐end anastomosis is inappropriate,a graft reconstruction can be considered. Autologous tissue is accepted to be the ideal alternative material due to its special advantages, namely that there is no immune rejection after implantation. More importantly, the autologous tissue can be completely integrated into adjacent areas without apparent inflammation, which can avoid some unpleasant complications, such as infection, dehiscence, and mobilization. Autologous tissue transplantation is suitable for long‐segment noncircumferential tracheal defects or large‐scale defects. Researchers have conducted transplant evaluation and reconstruction attempts on various autologous tissues for decades, including costicartilage,^38^ omentum, muscle pedicle flap, pericardium, cartilage, and so forth. However, there is still no consensus on the optimal tissue to be transplanted. However, autologous tissue alone may sometimes fail to achieve physiological tracheal rigidity and the support of scaffolds may be required.

### Pedicle muscle flap

Pedicle muscle flaps are mainly applicable to noncircumferential airway defects with a scale of more than 4–6 cm that are not suitable for adoption of end‐to‐end anastomosis, including tracheoesophageal fistula, bronchopleural fistula, trauma, and so forth.^14^, ^15^, ^26^ Generally, a defect <2 cm can be sutured directly;^39^ however, the extent of tracheal involvement should not exceed one third of the perimeter of the tube to ensure sufficient ventilation of hilum after anastomosis.^40^ Moreover, the length of pedicle muscle flaps cannot be more than twice the width in order to maintain an adequate blood supply. Chest wall muscles are well‐known for their abundant blood supply and are one of the most familiar pedicle flaps in clinical practice. Desai et al. once combined absorbable poly mesh with the pectoralis major muscle to cover the anterior tracheal defects.^39^ In addition, Ris adopted latissimus dorsi and serratus anterior transposition for 13 cases of reconstruction from 1996 to 2001, and found the respiratory pseudostratified ciliated epithelium overlying the muscle flap under bronchoscopy without any relative complications.^14^ Previously, chest wall muscle transposition has also been reported, and is adopted to repair the bronchial defect of the residual end, bronchopleural fistula, tracheoesophageal fistula after pneumonectomy.^41^, ^42^ In recent years, some novel techniques have been developed by using free fasciocutaneous flaps such as forearm free flap, with the complement of silicone scaffolds and the implantation of costal cartilage to strengthen the mechanical rigidity. The intraoperative revascularization, depending on surgical techniques, can be achieved by microanastomosis between the radical vessels of the flap and the small vessels of the neck under a magnifying microscope.^15^

### Pericardium flap

The pericardium flaps are always adopted in airway repair. In 2009, Brown et al. reported the treatment of long‐segment tracheal stenosis by pericardial patch, in which the trachea was cut along the midline at the site of stenosis and then the pericardium was implanted. There were no postoperative death cases associated with airway stenosis and no occurrence of relative syndromes in survival patients. In the following 3 months after the operation, the patch can be integrated into adjacent tissue perfectly.^43^ In fact, pericardium flap implantation has been used in clinical practice as early as 1984 when anterior pericardial tracheoplasty was designed to treat long‐segment stenosis and was once demonstrated to be able to dilate the airway by 1.5 times, which can satisfy the normal breath in the physical condition.^44^, ^45^ The survival rate was up to 92% and all the survival patients had no symptoms during the follow‐up period. In 2002, Fica et al. adopted a novel technique with a pericardium flap to treat a tracheal hamartoma. During this operation, they raised a pericardial flap that was kept attached to the aortic root and passed it under the brachiocephalic arterial trunk to the right side of the trachea, followed by a continuous suture.^46^

### Omentoplasty

The greater omentum contains more fat and is rich in blood vessels, lymphatic vessels, and nerve tissue. It is characterized by great extensibility, strong anti‐inflammatory ability, a high success rate of transplantation, and a tendency to adhere to other tissue leading to forming rich vascular anastomosis, which make the omentum become a talent in tissue repairing. Therefore, the greater omentum, in the form of a pedicle to preserve the blood supply or wrapped on the surface of the graft, has been proven to be effective to cover the defect and for local function to be recovered.^47^, ^48^, ^49^ The application of an omentum flap to repair the defect can reduce anastomotic complications. Actually, the characteristics of the greater omentum in the reconstruction of other organs (such as the kidney, gastrointestinal tract, bladder, and brain) have previously been investigated. However, the harvest of omentum will be bound to cause potential damage and sometimes intestinal obstruction due to its intraperitoneal operation.

### Other materials

Costal cartilage is often employed as an accessory to enhance the mechanical strength of the implant and plays a supporting role similar to that of tracheal cartilage. In addition to costal cartilage, there is also nasal septum cartilage and other cartilages. However, these materials are more commonly used in tissue engineering as a source of preseeded cells.

## ALLOGRAFT TRANSPLANTATION

Although it has been demonstrated that autologous tissue (such as costicartilage, pericardium, and muscle flap) has considerable potential to cover tracheal defects, they cannot be employed to reconstruct circumferential airway defects. Therefore, it is necessary to seek a new alternative in tracheal reconstruction. Although allograft tissue reconstruction can be selected as an option, it has two obvious drawbacks. On the one hand, the tissue pertains to allogenic tissue, which will inevitably cause immune rejection. Therefore, acceptors are required to take immune inhibitors. However, researchers have recently discovered many measures to reduce the immune response, such as acellular treatment and cryopreservation, which could significantly improve the success rate of transplantation. On the other hand, the graft is heavily dependent on donors, mostly from fresh corpses, which provokes some ethical disputes.

Aorta is one of the most popular grafts and has received wide attention. As is recognized, the aorta is characterized by relatively low immunogenicity, less vascularity, no secretion, and a stable nature. Allogeneic aortic transplantation was first reported in 1999 by Carbognani et al. on rabbits, in which an aortic graft was wrapped by omentum and implanted with a silica gel stent. Surprisingly, these rabbits started revascularization 7 days after the operation.^50^ Other than omentum, some researchers also employed chest muscle flap to promote revasculation.^51^ Since then, many experiments have been performed and all of them have confirmed the advantages of the aorta. In 2003, Martinod et al. replaced the trachea in sheep with aorta 5 cm in length. The results showed that the aorta was gradually transformed into the trachea from 6 months after the operation and the transformation was not completed until 6 years after the operation.^52^ To conduct further studies, this team increased the length of the replacement by 8 cm in 2005. The results showed that only one out of 20 patients died with no complications, and the anatomy confirmed that the aorta was completely transformed into a tracheal structure.^53^ Moreover, the studies by Seguin et al. in 2006^54^ and Anoosh et al. in 2009 on dogs^55^ also reached similar conclusions.

Although aortic transplantation possesses certain advantages, it also has some potential drawbacks, such as immune rejection and heavy dependence on donors. In addition, most aortic transplantation needs temporary or permanent scaffolds to prevent airway collapse, as the aorta is somewhat soft and lacks a certain rigidity. Almost all aortic grafts are implanted with stents, most of which are silicone scaffolds. An immune response is inevitable and has been a huge challenge in transplantation, although the aorta has less immunogenicity. In this regard, acellular treatment and cryopreservation are the most common methods to reduce immunogenicity. Cryopreservation can destroy endothelial cells, which play a major role in immune response. Therefore, this graft does not require immune inhibitors and can be preserved permanently. In contrast, the incidence of complications through acellular treatment is relatively higher and epithelial transformation is much lower than that of cryopreservation.^56^ However, frozen aortic transplantation trials on pigs by Marquette et al. in 2010 showed contrary results, namely that the severe inflammation occurred 3 months after the operation, and more than 60% of pigs had moderate or severe stenosis. However, all pigs were finally confirmed to have chondrogenesis.^57^ Hence, further investigations and animal trials to explore the measures to decrease the aortic immunogenicity are warranted.

Allotracheal transplantation once prevailed in the 20th century, but it stalled due to the scarcity of donor sources. Some scholars have conducted abundant studies before the invention of tracheal transplantation. The first endeavor has been to explore autologous esophageal transplantation with the assistance of its trachea‐like tubular structure, but the results have been disappointing. The first case of esophageal replacement, in 1962, died of pneumonia and apnea 6 weeks after the operation.^58^ In 1971, Fonkalsrud performed a similar operation. Unfortunately, 39 days after the operation, the patient suddenly died of intratracheal mucus blockage after experiencing severe respiratory distress, which blocked the application of anastomosis.^59^ These cases demonstrate the irrationality of esophageal transplantation, for the fact that the esophagus lacks ciliated cells and their clearing function like that of the trachea, which results in serious complications. Soon, the study of allogeneic tracheal transplantation commenced. The team of Rose et al. performed the first tracheal transplantation in 1979, in which they implanted the trachea harvested from fresh corpses into sternocleidomastoid in a patient in the first stage and the next stage was to reimplant the trachea covered by the pedicle muscle flap into the body of the patient, who was subsequently discharged from hospital without any complications. The success of this operation confirmed the feasibility of tracheal transplantation. However, it remains puzzling that the patient did not receive any immunosuppressive measures or other treatment of the trachea, and did not present with any immune rejection.^60^ Although tracheal transplantation, a potential substitute for trachea transplantation, can restore the physiological structure and function of the original trachea to a large extent, its clinical implementation is still limited due to the shortage of donor sources and the subsequent discovery of immune rejection.

## BIOENGINEERING

Bioengineering is an emerging field and research hotspot. Based on bioengineering, living cells are employed to expand to a certain scale in vivo and in vitro. In addition, biosynthetic materials or polymer frameworks are introduced to form alternative tissue as a result of an ideal solution to overcome the shortage of transplant tissue. Bioengineering first began to develop in 1933. However, there are extensive controversies over bioengineering in ethical and other aspects. In addition, in these decades no consensus has been reached on the four crucial elements of bioengineering, including cells, scaffolds, bioreactor, and growth factors.

An ideal tracheal substitute should be transversely flexible and longitudinally rigid to provide sufficient mechanical strength and avoid collapse. Moreover, some complications, such as respiratory distress and obstructive pneumonia, should be reduced to some extent. In addition, the implanted substitute should be tight (including air‐tight as well as liquid‐tight properties), and have favorable biocompatibility to be integrated into the surrounding tissue. The materials of the graft should be inert, nontoxic, nonimmunogenic, noncarcinogenic, durable, and nonmobilizable.

### Major challenges in bioengineering

The primary challenge of tissue engineering should be the obtainment of living cells. The major concerns involve the optimal kind of cells to be harvested and the appropriate method for the obtainment. In the past several decades, most attempts at bioengineering failed within a few months owing to recurrent infection and delayed revascularization.^61^ In the initial stages after implantation, the survival of the graft depends on the infiltration of interstitial fluid from surrounding tissue and nutrition derived from external vessels which are gradually extended into implanted regions. However, this type of revasculation is very slow, and would take several weeks to months. Further, the substitute will have risks of ischemic necrosis and ischemia–reperfusion injuries during this period.^62^ Delaere et al. concluded that it would take nearly 4 months to construct a complete revascularization system after the transplantation of allograft trachea following successful indirect revascularization.^63^ As peripheral vessels penetrate the transplanted trachea from the upper and lower anastomosis, a few months would be required to cover the midpoint of long‐segment tracheal substitute, which would result in local inflammation and stenosis due to the necrosis in the central region.^64^ Certainly, such complications would not occur in the short‐segment trachea due to the fact that endothelial cells from the natural trachea proliferate and then grow into the graft to establish an instant blood supply. In addition, the degradation rate of scaffolds should match the generation rate of new tissue. If the scaffolds degrade too fast, it will cause instability of the graft, combined with mobilization, leakage, inflammation, and formation of new tissue. On the contrary, the excessively slow degradation rate will hinder the generation and growth of new tissue. Moreover, the bioengineered trachea is exposed to the outside surroundings while the ciliated epithelium has not formed in the early stage. For that reason, the secretion and microorganisms which adhere to the tracheal surface cannot be removed, which will usually induce inflammation as well as infection. The mechanical strength of tracheas is another impediment. The excessive rigidity will reduce its ductility and flexibility, while the excessively low rigidity will lead to collapse, obstruction, respiratory distress, and even death. In addition, immune rejection and the supply of oxygen and nutrients are also crucial challenges that cannot be neglected.

## CONCLUSIONS

There are still many challenges for tracheal and carinal reconstruction. In addition, relevant standards or guides are still insufficient. Although many new technologies have been adopted to improve the prognosis and survival of patients, the most important things still involve the determination of qualified patients and the development of precise manipulation. With the continuous advancement and improvement in technologies, tracheal reconstruction will gradually become mature. It is definitive that bioengineering will be the tendency of tracheal surgery in the future, and it is currently developing in the right direction. However, the real significance of this technology is still unclear together with its impact on tracheal reconstruction. Surgical reconstruction is still irreplaceable, and it is the most effective therapy for the treatment of tracheal defects. It can be expected that the absolute replacement of tracheas will be realized in the future, which will benefit all individuals with tracheal diseases.

## CONFLICT OF INTEREST

None declared.
